# Distinctive Traits of European Mistletoe (*Viscum album* spp. *austriacum*) and Its Impact on Host Tree Wood (*Pinus sylvestris*)

**DOI:** 10.3390/plants14101489

**Published:** 2025-05-16

**Authors:** Alicja Dołkin-Lewko, Esra Pulat, Roman Wójcik, Barbaros Yaman, Urszula Zajączkowska, Tomasz Oszako, Mirela Tulik

**Affiliations:** 1 Department of Forest Botany, Warsaw University of Life Sciences, 02-787 Warsaw, Poland; urszula_zajaczkowska@sggw.edu.pl; 2 Department of Forest Botany, Bartin University, 74110 Bartin, Turkey; epulat@bartin.edu.tr (E.P.); byaman@bartin.edu.tr (B.Y.); 3 Department of Forest Management Dendrometry and Economics of Forestry, Warsaw University of Life Sciences, 02-787 Warsaw, Poland; roman_wojcik@sggw.edu.pl; 4 Department of Forest Protection, Forest Research Institute, 05-090 Sękocin Stary, Poland; t.oszako@ibles.waw.pl

**Keywords:** forest dieback, callose, structural dimorphism, parasite–host interaction, wood

## Abstract

European mistletoe is a hemi-parasitic plant increasingly infesting forests in Central Europe, causing premature tree death, and is anticipated to expand its range due to global warming. This study aimed to describe the unique anatomical features of mistletoe and examine the morpho-anatomical response of pine trees to infestation. Anatomical analyses were conducted on mistletoe internodes and the branch wood of affected pines. The findings revealed that mistletoe infestation triggers callose deposition in the cell walls of pine tracheids, a defense mechanism that restricts water flow to the mistletoe. Unique structural features of mistletoe were also identified, including structural dimorphism with the inner system forming only vessels and parenchyma cells, in contrast to the outer system, composed of protective, ground, and conductive tissues, and which displays an uneven distribution of chlorophyll and starch grains along the plant axis. Additionally, starch and chlorophyll were present in the parenchyma cells of the haustorium. Starch presence there may potentially enable internal photosynthesis, and the compounds formed after starch hydrolysis may facilitate water uptake from the host’s xylem sap. These results provide new insights into the anatomical adaptations of mistletoe and the defensive responses of pine trees, contributing to a deeper understanding of host–parasite interactions in forest ecosystems.

## 1. Introduction

In recent years, the number of key forest-forming species has been declining due to the impact of biotic agents [1,2,3,4]. Mistletoes are a significant factor in the weakening health and premature dieback of trees. They are often considered predisposing agents to the dieback of both deciduous and coniferous trees by increasing drought stress in the host, particularly in xeric sites [5,6,7,8]. Mistletoes are common in the temperate zone, dry regions, and humid tropics [9] but they are not found in extremely cold regions [10]. As a hemi-parasitic plant (hereinafter for simplicity it will be called a parasitic plant), mistletoe attaches itself to a tree and develops an intrusively growing organ that comes into contact with the host wood and relies entirely on water, inorganic nutrients, and host carbon, although it is partially capable of fixing atmospheric carbon [11,12,13]. Marshall et al. [14] and Lüttge et al. [15] showed that 24% to 62% of the carbon in mistletoe is of heterotrophic origin. In this way, mistletoe modulates the functioning of the host, e.g., at the level of its physiology. Hosts affected by mistletoe must adapt their hydraulic system to limit water leakage, which closes the stomata and reduces the rate of transpiration and assimilation [16,17]. In contrast, mistletoe always has a higher transpiration rate and stomatal conductance, which is suggested to help access to nutrients such as nitrogen from the host’s wood [12,18,19,20], and the adoption of a more negative water potential. In response to mistletoe infestation, host plants tend to decrease leaf area/sapwood area ratios [21,22] and reduce the rate of photosynthesis due to leaf nitrogen depletion [14,21,23]. These effects can cause or exacerbate C starvation and host mortality [6,24]. Hu et al. [25] found a change in the phytohormone profile and anti-oxidative metabolism in the bark and wood of the host caused by mistletoe infestation as well. The impact of mistletoe on trees also manifests itself in the deformation of infected trunks or branches, reduced growth and vigor, and the production of less wood of poor quality [26,27,28]. Srivastava and Esau [29] observed a significant increase in the shape and size of infested rays, which was the effect of dwarf mistletoe on the anatomy of conifer wood. The axial tracheids were shorter, wider, and more irregular in shape than in healthy wood. Furthermore, the authors observed a larger number of resin canals than those from healthy trees. In turn, Ozturk et al. [30] investigated the quantitative characteristics of pine wood and showed that the most pronounced changes were related to the reduction in the double wall thickness, lumen area, tangential lumen area, and radial lumen area of the tracheids.

Recent results have also shown that mistletoe can affect the reproductive success of its hosts, as parasitized individuals produce fewer fruits and have reduced seed weight [31,32,33,34]. Additionally, in response to adverse factors, mistletoe may absorb host defense chemicals (secondary metabolites and hormones) and use them in its defense [12,35] and to complete its life cycles [23,36,37,38].

The activity of mistletoe is also manifested by changes at the tree population level, which leads to a modification of the structure and dynamics of the forest ecosystem. This hemi-parasite reduces host productivity [21,28,39,40] and can indirectly change the competitive balance between host species and other species in the community [41] and the quantity and quality of resources introduced into the soil [42]. However, these modifications may be more difficult to detect due to the long lifespan of the ecosystem [43]. In addition, host mortality caused by mistletoe can lead to open gaps [44]. European mistletoe (*Viscum album*) is distributed throughout Eurasia from Great Britain to northern Asia and is a known bird-borne plant, with pharmaceutical potential, and a symbol in mythology [45,46]. The anticancer activity of several compounds isolated from its leaves has been confirmed in clinical trials [47]. Nowadays, *V. album* is harvested for Christmas decorations. It prefers a mild oceanic climate with summer temperatures above 15 °C and in winter not less than − 7 °C. Climatic conditions are key factors influencing the growth and distribution of *V. album* [48], and its range is also influenced by the presence of a suitable host [45,49].

Scots pine (*Pinus sylvestris* L.) is widespread in the boreal and temperate zones of Eurasia, but its distribution is predicted to decline [50]. Scots pine is the main host of European pine mistletoe (*Viscum album* L. ssp. *austriacum* (Wiesb.) Vollm), whose body is made of two systems: the external one (outer) consisting of pseudo-dichotomously branching evergreen shoots and the internal (inner) system represented among others by an absorbing organ called the haustorium (sinker) that penetrates the host’s tissue [51]. Its fruits are yellow.

There is no information in the literature on the developmental anatomy of the shoot of *V. album* spp. *austriacum*. Some papers contain data on the anatomy of the aerial organs of this species or other species of the *Viscum* genus [52,53]. These authors described the anatomy of the one-year-old shoot as typical for dicotyledonous plants and stated that the shoot structure is similar in *Viscum* taxa in terms of epidermis cells, thick cuticle, stoma types, starch grains, and oxalate crystals.

The geographic range of *V. album* spp. *austriacum* covers the northwestern part of Africa (Morocco), the Iberian Peninsula, Central and Central Southern Europe, the western part of Eastern Europe, the Caucasus, and Asia Minor [49].

Since mistletoe infestation of pines tends to increase as a result of ongoing climate warming [24,49,54] and no work has been published on the developmental anatomy of mistletoe, this work aims to (i) describe the unique anatomical features of mistletoe and (ii) indicate the pine’s response to the influence of mistletoe at the morpho-anatomical level. In analyzing the unique anatomical features of mistletoe, we focused, among other things, on the location of starch within its cells because starch is thought to be stored for use on different time scales, and its metabolism also depends on environmental conditions [55]. In addition, starch is a key molecule in mediating plant responses to biotic and abiotic stresses, such as water deficit, high salinity, or extreme temperatures. Under unfavorable environmental conditions, plants typically remobilize starch to provide energy and carbon during periods when photosynthesis may be impaired [56]. Starch is also considered to play a crucial role in gravity sensing according to the statolith theory [57,58], although a series of studies on starchless mutants have indicated that starch is not absolutely essential but does play a role in sensing gravity and is necessary for a full gravitropic response [59,60,61]. In plants, two types of starch are present, i.e., transitory starch, which is usually synthesized and degraded within a day, in contrast to long-term starch, which is stored in amyloplasts to support vegetative or reproductive growth, reproduction, or stress response [62].

The aim of this study, among others, was achieved by analyzing the anatomy of subsequent internodes of mistletoe shoots and the branch wood of pines.

We hypothesized the following:

1. The pattern of growth and body organization of the outer system of *V. album* differs from those of the inner one.
2. By adopting the structure–function concept in plants, we postulate that host trees develop structural and physiological adaptations to mistletoe infestation, which is visible at the level of host wood anatomy, especially concerning wood qualitative features.
3. Starch is stored in the cells of the internal system of mistletoe, and although it is osmotically inert, after hydrolysis it becomes a source of solutes (e.g., glucose) that affect the water potential of mistletoe cells. The reduced water potential of mistletoe cells favors the uptake of water from the host’s xylem sap.

We believe that only a comprehensive understanding of the developmental anatomy of the shoot structure of *V. album* spp. *austriacum* and its relationship with the host can contribute to the development of effective methods to limit the spread of mistletoe in pine stands.

## 2. Results

**Figure 1:**
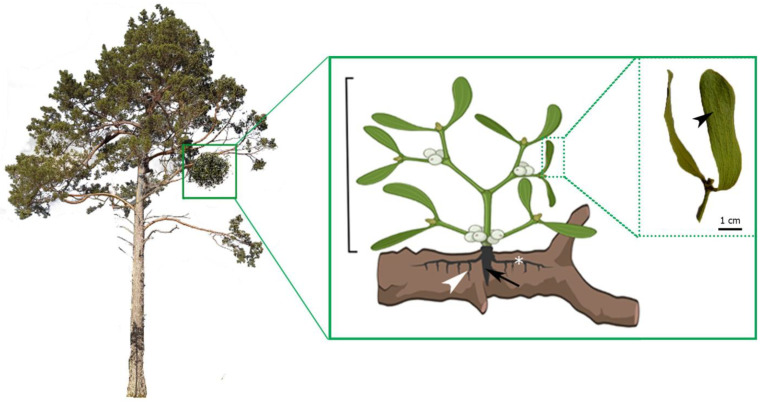
The scheme of the mistletoe body plan consisted of an external system (marked with a bracket) and an internal system with a haustorium (black arrow), sinkers (white arrowhead), and cortical strands (asterisk). Macroscopic inset view of a mistletoe leaf with parallel veins indicated by a black arrowhead.

Regardless of whether the mistletoe grew following the gravity vector or in the opposite direction, its structure consisted of a well-developed external system (outer system) and an internal system (inner = endophytic system). The internal system was in the form of the primary haustorium, sinkers (secondary haustorium), and cortical strands (Figure 1).

The shoot system had a bifurcated structure, with every annual increment represented by an internode bearing two opposite leaves with parallel veins (Figure 1). This type of venation is characteristic of monocotyledonous plants.

The leaves supported the bud that formed the following year’s increment. We found that the number of internodes did not determine the age of the mistletoe because the morphological age did not reflect the age of the parasite calculated based on the number of rings of the host’s wood infested with the primary haustorium.

**Figure 2:**
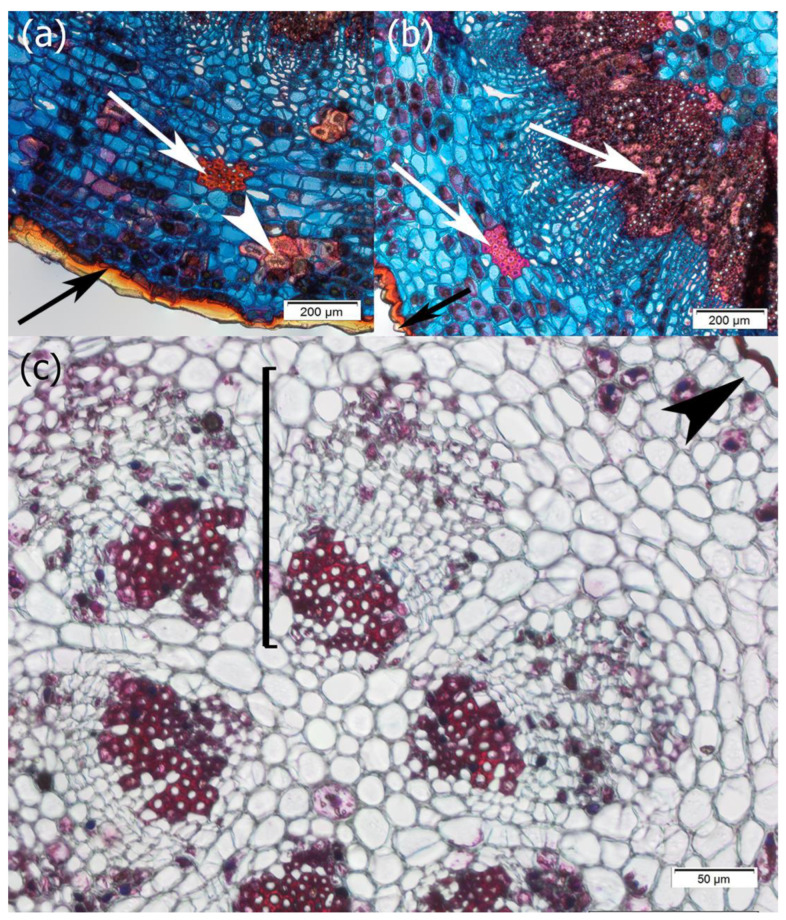
Cross-sections of mistletoe internode of shoot growing against the force of gravity. (**a**,**b**) Study samples were taken in the winter season of 2022 and (**c**) in the summer season of 2022. The sections were stained with FCA. Visible is the thick cuticle covering epidermal cells (black arrows), sclereids (white arrowhead), and fibers (white arrows) in the cortical tissue. The anticlinal division in the epidermal cell is highlighted with a black arrowhead. A bracket marks one of the vascular bundles.

**Figure 3:**
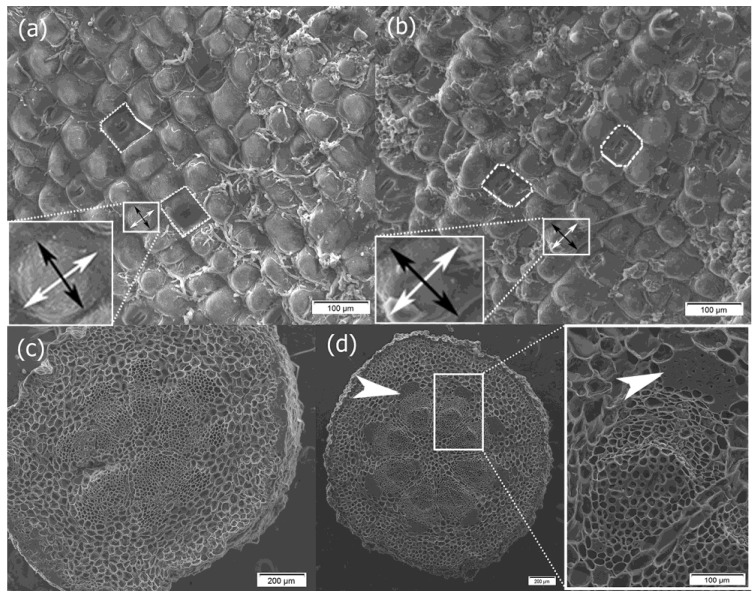
SEM. The internode surface of (**a**) one and (**b**) six years of age with epidermal cells arranged in longitudinal files and outlined paracytic stomata. White arrows indicate the axial direction and black arrows the transverse direction. The cell dimensions were measured for 10 randomly selected pavement cells. Cross-sectional surface of (**c**) one-year- and (**d**) two-year-old internodes. The vascular bundle cap is indicated by arrowheads.

The youngest internodes had a typical structure also observed among other dicotyledonous plants; that is, they were covered with a single-layer epidermis, the outer periclinal wall of which was thick and covered with a thick waxy waterproof cuticle (Figure 2a,b). The secretion of a highly hydrophobic lipid-rich cuticle into the thickened external cell wall matrix demonstrates the polarity of epidermal cells. The epicuticular wax usually appeared in the form of granules or irregular platelets according to the classification of Barthlott et al. [63]. The epidermis comprised pavement cells, which formed the bulk of the tissue, and guard cells, which controlled gas exchange and were surrounded by subsidiary cells (Figure 3a,b).

Both pavement cells and stomatal cells exhibited a characteristic pattern of distribution (Figure 3a,b). More or less square epidermal cells with a transverse dimension of 31.31 µm (SD 3.64) and a longitudinal dimension of 32.15 µm (SD 7.18) were arranged in linear rows along the axis of the internodes, while the stomata had a transverse orientation and were mostly of the paracytic and rarely anomocytic types (Figure 3a,b).

**Figure 4:**
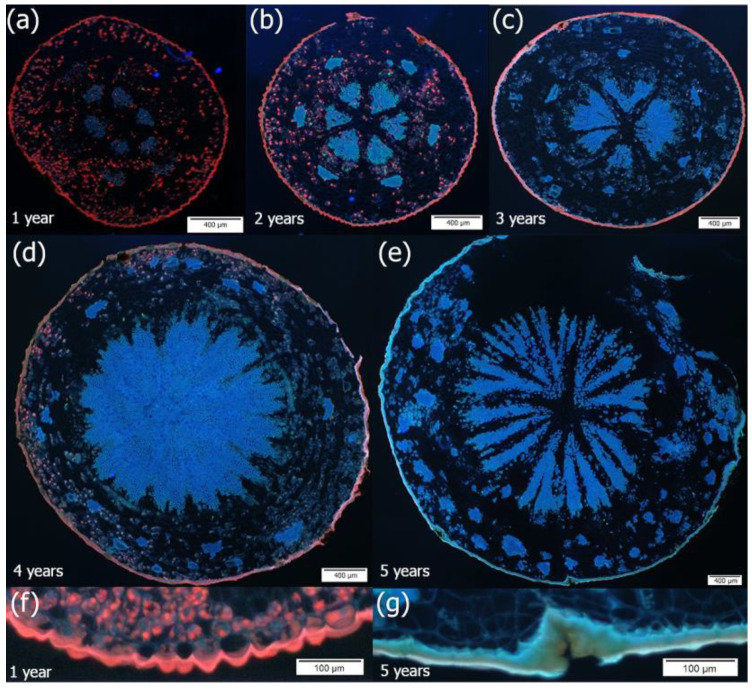
Cross-sections of the internodes of mistletoe aged 1 to 5 years. The shoot grew against the force of gravity. Red autofluorescence of asymmetrically distributed chlorophyll in cortex parenchyma cells is visible. (**a**–**e**) Secondary growth is based on the activity of the vascular cambium; (**f**) epidermis with thick cuticle covering 1-year-old and (**g**) 5-year-old internodes. Samples were taken in the summer of 2022. The sections were stained with aniline blue and observed under UV light.

**Figure 5:**
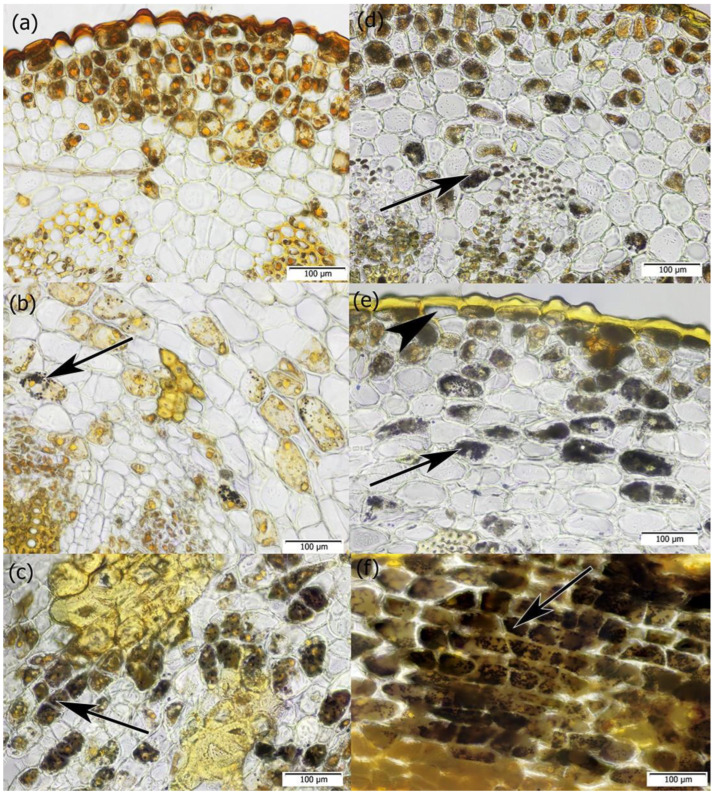
Cross-sections of the internodes of mistletoe. The shoot grew upward against gravity (**a**–**c**). The shoot grew in the direction of the force of gravity (**d**–**f**). The starch grains distributed asymmetrically (purple-black color) are marked with arrows in the internodes, from (**a**,**d**) the youngest internode to (**c**,**f**) the oldest internode. The black arrowhead indicates a circumferentially stretched epidermal cell. Samples taken in summer 2022. Sections stained with Lugol’s reagent.

Beneath the epidermis were elements of the primary cortex: parenchyma cells with chlorophyll, sclereids, and fibers (Figure 2a,b and Figure 4). The parenchyma cells also contained starch grains, which were observed both in samples collected in winter and summer periods (Figure 5).

In the central part of the one-year-old internode, there was a stele with open collateral vascular bundles and the interfascicular parenchyma between them. One cap of the vascular bundle was mainly attached to the primary xylem and the other cap above the primary phloem, but the full development of the xylem fiber cap ended in 2-year-old internodes (Figure 3c,d and Figure S1a–d). Secondary growth started from the procambium found in the bundles and through dedifferentiation of the cells of the interfascicular parenchyma, as is typical of woody seed plants (Figure 4). Secondary growth occurred in subsequent years only because of the activity of the vascular cambium, as the epidermis has a protective function throughout the lifespan of the mistletoe, never being replaced with periderm (Figure 4). However, anticlinal divisions were observed in the epidermal cells, which contributed to an increase in the number of cells in the tissue cylinder (Figure 2c). Moreover, as the stem circumference enlarged due to the activity of the bifacial vascular cambium, the epidermal cells increased in size and, for example, in the sixth internode, their transverse dimension was 59.09 µm (SD 7.60) and their longitudinal dimension was 61.71 µm (SD 8.79). This means that over 5 years, they increased their transverse dimension by 27.78 µm and longitudinal dimension by 29.56 µm. Nevertheless, among the epidermal cells, some were also circumferentially stretched (Figure 5e).

When we analyzed the presence of chlorophyll and starch grains in the parenchyma cells along the axis of successive internodes, it was observed that chlorophyll decreased in the basipetal direction while starch content increased. The deposition of chlorophyll and starch occurred in a polar manner, irrespective of whether the mistletoe shoot grew upward against gravity or in the direction of the force of gravity (Figure 4 and Figure 5).

**Figure 6:**
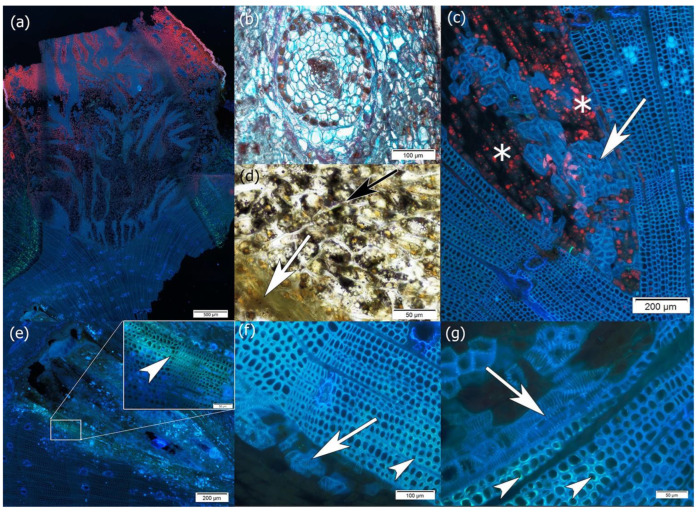
Cross-sections of infested tissues of a pine tree. (**a**) Host branch tissues infested with mistletoe—general view; (**b**) cross-section through a cortical strand, visible as a circular structure; (**c**) wood of branch with haustorium composed of vessels (white arrow) and parenchyma cells (asterisk), with chlorophyll showing red autofluorescence under UV light; (**d**) fragment of haustorium embedded in branch wood showing parenchyma cells with starch grains (black arrow) and vessels (white arrow); (**e**,**g**) white arrowheads mark host compression-like tracheids with yellowish green callose fluorescence in the cell wall; and (**f**,**g**) mistletoe vessels with spiral thickening of their cell walls placed along the edge of the haustorium, marked with white arrows. Samples taken in summer 2022. Sections stained with aniline blue and observed under UV light (**a**,**c**,**e**–**g**). (**b**) FCA and (**d**) Lugol’s reagent staining were also used.

At the site of mistletoe infestation, where a dome-shaped holdfast was formed, branch thickening was observed, and haustorium, sinkers, and cortical strands (linear endophytic structures developing within the host cortex) were present along the longitudinal axis of the host branch (Figure 6a,b). Haustorium starts from the adhesive disk and, after contact with the host’s vascular cambium, grows into its wood. Similarly to the haustorium, cortical strands that reached the host’s vascular cambium developed into sinkers, whereas those that remained within the cortex appeared in transverse sections as circular structures (Figure 6b). Only in their structures were phloem elements present (Supplementary Materials Figure S1e,f).

Unlike the external shoot, the haustorium and sinker wood were made only of vessels whose walls had spiral thickening and parenchyma cells containing chlorophyll and starch grains (Figure 6c,d).

The vessels of the haustorium/sinkers that reached the host vascular cambium were mainly located along the margin near the host tracheids, thus constituting the continuity of the host–parasite wood (Figure 6e–g). The host wood tracheids had a callose in the secondary cell wall layer, indicating compression wood features (Figure 6e–g).

Although resin ducts are a constitutive feature of pine wood, these structures were mainly observed near the haustorium and sinkers (Figure 6c,f).

## 3. Discussion

Nowadays, mistletoe (*V. album* spp. *austriacum*) is the subject of research due to its unique parasitic relationship with its main host and its role in forest ecosystems.

It is known that mistletoe spreads by sticky seeds, which are dispersed mainly by birds. Once the seed attaches to a host, it germinates and produces haustorium and sinkers—specialized organs that open up the host’s tissues and reach its vascular cambium and then the wood. Later, the elements of the external system develop. For *V. album*, the time required for the development of the first internode is 2 to 4 years, as reported by Heide-Jørgensen [51], or 4–5 years, as documented by Zuber [45]. This means that the age of mistletoe can be expressed by the number of internodes, i.e., its morphological age, or by the number of infested ring increments in the host wood, i.e., its anatomical age. In these studies, the difference between the morphological age and the age calculated for the haustorium (anatomical age) was two years.

Furthermore, the epidermis covers the shoot surface during the lifespan of mistletoe, and secondary growth is only realized with the activity of the vascular cambium. According to the tensile skin theory, the shoot epidermis plays a key role not only in protecting plants against dehydration and pathogens but also in ensuring proper organogenesis and growth control [64,65]. Savaldi-Goldstein et al. [66], based on experiments with brassinosteroids and Arabidopsis plants, showed that brassinosteroid signaling from the epidermis is not sufficient to establish normal vascular organization, but shoot growth is reduced when brassinosteroids are depleted in the epidermis. Therefore, they concluded that the epidermis both stimulates and limits shoot growth by providing a nonautonomous signal to ground tissues. Many studies have also indicated that cuticular lipids, similar to other lipid-related molecules, are involved in signaling during plant development [64]. Hence, the obtained results allow us to put forward the hypothesis that the mistletoe epidermis is the tissue that determines its lifespan.

We observed that stomata were located among the epidermal cells of the internodes. They were arranged transversely to the long axis of the organ, and this type of arrangement is not common in the plant kingdom. Butterfass [67] wrote that “transverse orientation is known from several mosses, from the Bennettitae, from the Azolla and some other ferns, and species of about 45 families of seed plants. It can also be confirmed that species of succulents exhibit this feature more frequently than other plants”. Our observations on the transverse orientation of stomata are consistent with the results reported by Khan et al. [68], who provided data on the anatomical characteristics of the organs of *Viscum*. We also conclude that the presence of stomata on the shoot surface ensures the high photosynthetic activity of this organ [13], especially since there are parenchyma cells containing chloroplasts with chlorophyll beneath the epidermis layer.

We found that chlorophyll and starch were deposited in a polar manner along the axis of the shoot, irrespective of whether the mistletoe shoot grew upward against gravity or in the direction of the force of gravity. Therefore, one may wonder, considering the starch–statolith theory [57,58], which states that starch grains act as statoliths that promote the organ’s response to gravity, whether low starch content in the apical part of the shoot contributes to the positive gravitropism of mistletoe shoots. The accumulation of starch is often correlated with the development of gravitropic competence [69]. However, the organs, such as the hypocotyl, flower stalks, and seedling roots in a starchless Arabidopsis mutant, are all gravitropic, which means that starch is unnecessary for gravity perception [59,60,61]. Considering the types of starch present in plant cells, i.e., transitory and storage starch [70], as well as starch source–sink relationships [62], we assume that transitory starch occurs mainly in the youngest internodes where it is rapidly degraded to provide carbon for developmental processes. In turn, the oldest internodes contain storage starch, which is produced in amyloplasts and is involved in gravity sensing. This suggests that diurnal starch turnover and sophisticated machinery that allows the organs to sense the direction of gravity and guide their growth should be studied in mistletoe in detail in the future. Two concepts can be tested: one based on the starch–statolith hypothesis and the other based on the protoplast pressure hypothesis (also known as the gravitational pressure theory), which assumes that the weight of the entire protoplast acts as the gravity sensor [71,72].

We also observed starch in the parenchyma cells of the endophytic system and proposed a function of starch stored in these cells as an example of metabolic adjustments. It is known that starch metabolism is very sensitive to changes in the environment [56,73]. This means that during unfavorable conditions, starch may provide osmoprotectants that favor water flow from the host toward the parasite. Pate et al. [74] reported cases in which the xylem sap concentrations of the host and the parasite (Australian mistletoes) were very similar, or in which the xylem sap of the mistletoe was four to five times more concentrated than that of the host. According to the latter finding, the selective accumulation of certain cations, such as potassium, allows for the maintenance of higher concentrations of these cations compared to host tissues, which was also observed by Wang et al. [75] in *V. album* spp. *album*. As a result of the accumulation of osmotically active substances, the transpiration of mistletoe is always greater than that of the host branches, which significantly reduces the efficiency of water use by the host tree [16,25] and is most dangerous during periods of drought when trees attacked by mistletoe suffer greatly from severe drought stress. In turn, chlorophyll noted in the haustorium and sinkers may indicate their participation in corticular/wood photosynthesis. The refixation of some of the CO_2_ respired by underlying tissues or carried to the branch/trunk segment by transpiration flux, which is potentially important for the carbon economy of trees and shrubs and helps to avoid the risk of an energy crisis due to low oxygen concentration, has been described in many reports [76,77,78,79]. Therefore, corticular/wood photosynthesis of mistletoe may be a way to maintain the C budget and avoid hypoxia/anoxia while developing inside the host tissues.

Apart from parenchyma cells, vessel elements whose walls were sculptured in the form of helical thickenings were observed in the endophytic system. Costa and Wiedenhoeft [80] investigated the structure–function relationships of helical thickenings and postulated three related roles, i.e., providing additional mechanical support, refilling vessels after cavitation, and increasing hydraulic efficiency. This suggests that helical thickening in the cell walls of mistletoe vessel elements may serve a protection function in the event of hydraulic failure.

Elements of mistletoe phloem were observed only in cortical strands, which appeared circular in the transverse section and were absent from the haustorium and sinkers. The haustoria of certain parasites, such as *Orobanche* and *Cuscuta* spp., form connections with the host’s xylem and phloem, as reported by Fisher et al. [42], which reflects their strong dependence on host photosynthesis. *V. album* spp. *austiacum* develops direct contact with the host’s vascular cambium and its wood. It appears to be photosynthetically self-reliant, with structural and physiological adaptations. The question, however, arises whether and how the phloem elements of the endophytic and external mistletoe systems are connected. Radioactive tracer experiments would be helpful for finding the answer to this question.

In the examined wood samples of the host, compression-like tracheids occurred on the upper side of pine branches, mainly along the edge of the haustorium, where mistletoe vessels appeared. Compression wood is a consequence of the geotropic response of conifers, *Taxus*, and *Ginkgo* and is usually found on the underside of an inclined trunk/branch [81]. In the case of the wood samples we examined, the gravitropic response can be ruled out. Compression-like tracheids in the wood of pines formed in polluted environments were also observed by Kurczyńska et al. [82]. Callose is a polysaccharide present in the cell walls of numerous higher plants. It plays crucial roles in various aspects of plant growth, development, and stress responses, including defense against pathogens and adaptation to biotic and abiotic stresses [83,84,85,86]. Therefore, we hypothesize that the anomalous presence of callose in the cell walls of host tracheids may indicate a pine tree’s response to mistletoe infestation, as callose is synthesized rapidly and deposited in a localized manner under different types of stress [87]. During fungal infections, callose is deposited in the cell wall beneath infection sites and is considered to provide a physical barrier to penetration [88,89]. It is also worth noting that compression wood is generally predisposed to perform a mechanical function [81], not a conductive one; therefore, the compression-like tracheids may also constitute a barrier zone hindering the flow of water from the host to the parasite, especially since water transfer between the host wood and the parasite usually takes place mainly in the apoplastic mode [90,91]. Additionally, loading the host branch with mistletoe is a mechanical challenge. Tree branches colonized by mistletoe were particularly prone to failure. Therefore, failure may be counteracted by the formation of compression-like tracheids that act mechanically. These changes in the structure of pine wood described above allow us to provide a hypothesis assuming that host trees develop structural and physiological adaptations to mistletoe infestation.

Although resin ducts are a constitutive feature of pine wood, these structures were more abundant near the haustorium and sinkers, which may also indicate a defensive reaction of pine to mistletoe infestation owing to the toxic properties of oleoresin. Srivastava and Esau [29], as well as Klutsch and Erbiling [92] and Ferrenberg [93], reported an increase in the number of resin canals and resin production in pine trees infested with dwarf mistletoe.

The results of this study allow us to accept our three hypotheses. Anatomical analyses confirmed the distinct organization of the internal and external systems of *Viscum album* ssp. *austriacum*, thereby supporting the first hypothesis. The presence of compression-like tracheides and the distribution of resin ducts are consistent with the assumption that host trees respond to mistletoe infestation on the morpho-anatomical and physiological levels. Accumulation of starch as a source of osmoprotectants in the endophytic system allows us to accept a third hypothesis. We believe that our findings can contribute to the development of effective methods to limit the spread of mistletoe in pine stands. Nevertheless, research showing infested tree response and unique structural features that favor mistletoe in attacking trees and extending its range still needs to be conducted.

## 4. Materials and Methods

Plant material in the form of fragments of pine branches (*P. sylvestris*) and mistletoes (*V. album* ssp. *austriacum*) anchored in tree branches was collected in January and June 2022, respectively. Every time, the material came from at least 2 infested branches of 3 different pine trees, which did not show visible symptoms of decline. Pines’ crown infestation reached about 50–70%.

The infested 80-year-old pines formed a community of moist coniferous forest in the Kozienice Forest District (51° 25′ 31” N, 21° 17′ 14” E), where a large area inventory of infested trees was carried out by Wójcik et al. [94]. The mistletoes growing vertically up and down in response to the earth’s gravitation pull were selected for the study, and their morphological age expressed by the number of internodes was 5–8 years.

Internodes of different morphological ages were cut from the external system of the mistletoe and, along with the absorbing organs of the parasite infecting the host wood, they were fixed in 70% ethanol and glycerin in a 1:1 ratio by volume. Both the internodes and haustorium with the host wood were cut using a vibratome VT l000S (Leica, Wetzlar, Germany) into cross-sections and longitudinal sections with a thickness of 30 µm. Then, a part of the obtained sections was incubated in the dark for at least 5–10 min in a solution of 0.1% (*w*/*v*) aniline blue in 0.15 M K_2_HPO_4_. The same sections were then placed in a drop of aniline blue and viewed with UV extinction (*λ_ext_. =* 365 nm) to detect callose in the tracheid cell walls of the host wood. In UV light, callose exhibits yellowish green fluorescence. Later sections of internodes, haustorium, and host wood were stained with Lugol’s starch grain detection reagent and stained according to Etzold [95] to distinguish lignified from unlignified cell walls. Observations were carried out under a Provis AX70 light microscope equipped (Olympus, Tokyo, Japan) with a UC90 digital camera (Olympus, Tokyo, Japan). The CellSens Standard 1.18 (SIS) software was used to acquire microscopic images. Images presented in the figures were uniformly adjusted for brightness and contrast to improve clarity, without altering the original data. Additionally, small samples of every internode were observed with a scanning electron microscope (FEI Quanta 200; Thermo Fisher Scientific, Waltham, MA, USA) to detect cuticle ornamentation. To indicate the developmental trend of pavement cells, their transverse and longitudinal dimensions were measured for 10 randomly selected cells in every internode. The obtained results were averaged, and the standard deviation (SD) was estimated.

## Data Availability

All data generated or analyzed during this study are included in this article and are available from the corresponding author upon reasonable request.

## Supplementary Materials

The following supporting information can be downloaded at https://www.mdpi.com/article/10.3390/plants14101489/s1. Figure S1. (a,b) Cross sections through 1-year-old mistletoe internodes. Lack of fully developed xylem fibers. (c,d) Cross sections through 3-year-old mistletoe internodes. The black arrows indicate fully developed xylem fibers. (e,f) Transverse sections through cortical strands running longitudinally within the host cortex, visible as circular structures; phloem is indicated by white arrowheads. Samples (a,b) were collected in the winter season of 2022, and (c–f) in the summer season of 2022. Sections were stained with FCA (a–d) and aniline blue and observed under UV light (e,f). Visible yellowish green fluorescence of callose in sieve pores is indicated by white arrowheads.
