# Supplementary material for: Distinctive Traits of European Mistletoe (Viscum album spp. austriacum) and Its Impact on Host Tree Wood (Pinus sylvestris)
Source: Plants (Basel). 2025 May 16;14(10):1489. doi: 10.3390/plants14101489 (PMC12114866; doi:10.3390/plants14101489)
Supplement: Supplementary file 1 [file plants-14-01489-s001.zip › plants-3502438-supplementary.pdf]

Supplementary materials

These supplementary materials provide additional anatomical evidence supporting the findings of the study. In particular, they illustrate changes in the structure of mistletoe internodes with age. Cross-sections show the progressive development of secondary xylem, including the appearance of fibers in older internodes. Additionally, transverse sections of cortical strands within the host cortex demonstrate the presence of phloem tissue, clearly visible under UV light in aniline blue-stained sections.

The images represent transverse sections of *Viscum album* ssp. *austriacum* internodes collected during both the winter and summer seasons of 2022. Sections were stained with either FCA (Fuchsin-Chrysoidine-Astra blue) or aniline blue, depending on the specific anatomical features being analyzed.

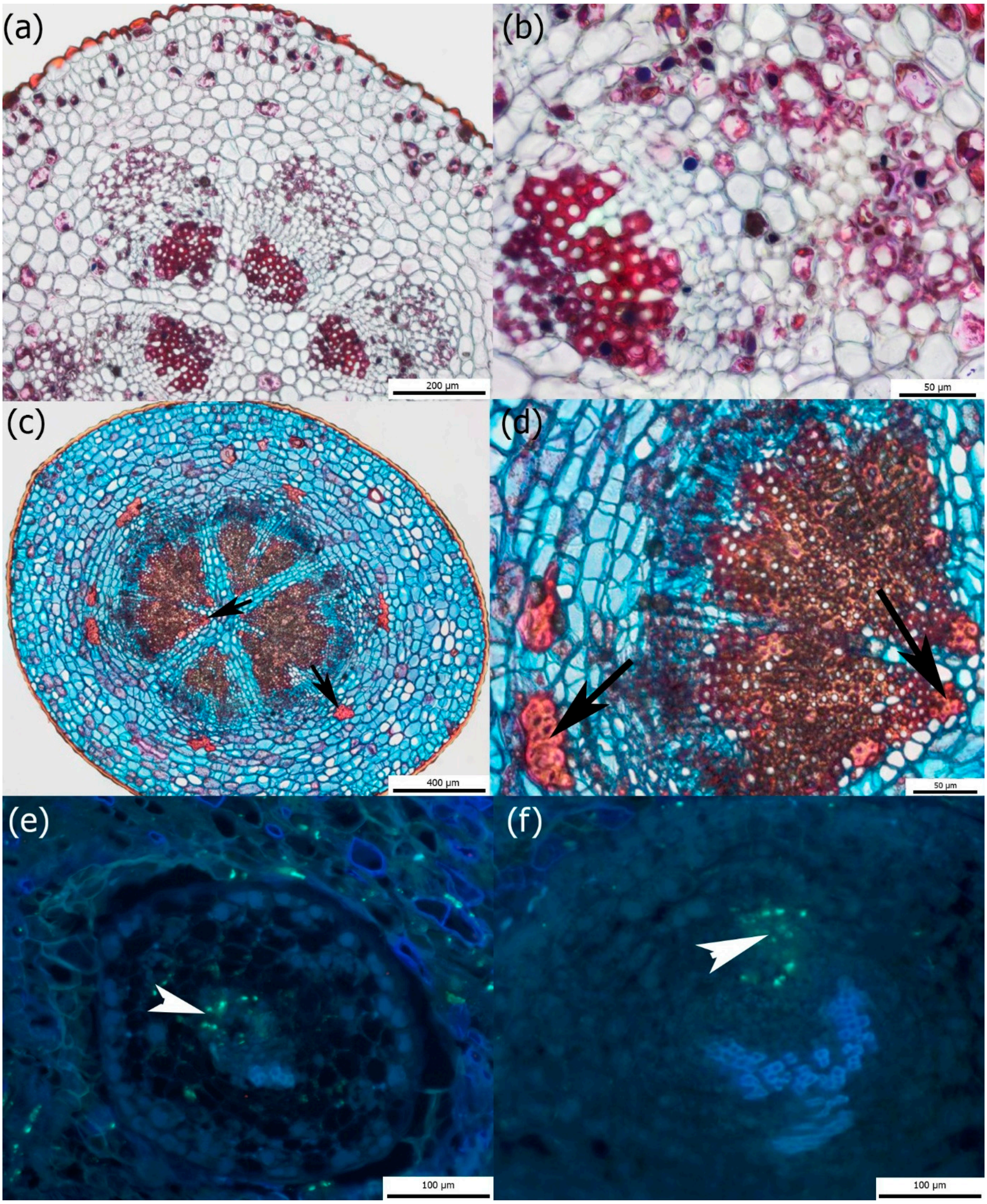

Supplementary Figure S1. (a–b) Cross sections through 1-year-old mistletoe internodes. Lack of fully developed xylem fibers. (c–d) Cross sections through 3-year-old mistletoe internodes. The black arrows indicate fully developed xylem fibers. (e–f) Transverse sections through cortical strands running longitudinally within the host cortex, visible as circular structures; phloem is indicated by white arrowheads. Samples (a–b) were collected in the winter season of 2022, and (c–f) in the summer season of 2022. Sections were stained with FCA (a–d) and aniline blue and observed under UV light (e–f). Visible yellowish green fluorescence of callose in sieve pores is indicated by white arrowheads.
